# Educational strategies in the health professions to mitigate cognitive and implicit bias impact on decision making: a scoping review

**DOI:** 10.1186/s12909-023-04371-5

**Published:** 2023-06-20

**Authors:** John Thompson, Helena Bujalka, Stephen McKeever, Adrienne Lipscomb, Sonya Moore, Nicole Hill, Sharon Kinney, Kwang Meng Cham, Joanne Martin, Patrick Bowers, Marie Gerdtz

**Affiliations:** 1 Department of Nursing, Faculty of Medicine, Dentistry and Health Sciences, The University of Melbourne, Level 6, Alan Gilbert Building, 161 Barry Street, Victoria, 3010 Australia; 2 Royal Children’s Hospital, Parkville, Australia; 3 Department of Physiotherapy, Melbourne School of Health Sciences, University of Melbourne, Melbourne, Australia; 4 Department of Social Work, Melbourne School of Health Sciences, University of Melbourne, Melbourne, Australia; 5 Department of Optometry and Vision Sciences, Melbourne School of Health Sciences, University of Melbourne, Melbourne, Australia; 6 Department of Audiology and Speech Pathology, School of Health Sciences, University of Melbourne, Melbourne, Australia

**Keywords:** Cognitive bias, Implicit bias, Tertiary education, Assessment, Healthcare education, Clinical decision-making

## Abstract

**Background:**

Cognitive and implicit biases negatively impact clinicians’ decision-making capacity and can have devastating consequences for safe, effective, and equitable healthcare provision. Internationally, health care clinicians play a critical role in identifying and overcoming these biases. To be workforce ready, it is important that educators proactively prepare all pre-registration healthcare students for real world practice. However, it is unknown how and to what extent health professional educators incorporate bias training into curricula. To address this gap, this scoping review aims to explore what approaches to teaching cognitive and implicit bias, for entry to practice students, have been studied, and what are the evidence gaps that remain.

**Methods:**

This scoping review was guided by the Joanna Briggs Institute (JBI) methodology. Databases were searched in May 2022 and included CINAHL, Cochrane, JBI, Medline, ERIC, Embase, and PsycINFO. The Population, Concept and Context framework was used to guide keyword and index terms used for search criteria and data extraction by two independent reviewers. Quantitative and qualitative studies published in English exploring pedagogical approaches and/or educational techniques, strategies, teaching tools to reduce the influence of bias in health clinicians' decision making were sought to be included in this review. Results are presented numerically and thematically in a table accompanied by a narrative summary.

**Results:**

Of the 732 articles identified, 13 met the aim of this study. Most publications originated from the United States (*n=*9). Educational practice in medicine accounted for most studies (*n=*8), followed by nursing and midwifery (*n=*2). A guiding philosophy or conceptual framework for content development was not indicated in most papers. Educational content was mainly provided via face-to-face (lecture/tutorial) delivery (*n=*10). Reflection was the most common strategy used for assessment of learning (*n=*6). Cognitive biases were mainly taught in a single session (*n=*5); implicit biases were taught via a mix of single (*n=*4) and multiple sessions (*n=*4).

**Conclusions:**

A range of pedagogical strategies were employed; most commonly, these were face-to-face, class-based activities such as lectures and tutorials. Assessments of student learning were primarily based on tests and personal reflection. There was limited use of real-world settings to educate students about or build skills in biases and their mitigation. There may be a valuable opportunity in exploring approaches to building these skills in the real-world settings that will be the workplaces of our future healthcare workers.

**Supplementary Information:**

The online version contains supplementary material available at 10.1186/s12909-023-04371-5.

## Background

Human judgement is inherently subjective, uncertain, and therefore, prone to bias [1]. In healthcare environments, errors in clinical reasoning can have a devastating impact on individuals and populations [2]. To mitigate risk of bias arising from cognitive and implicit influences, codes of conduct have been established to provide moral standards that guide clinical decision-making.

Egalitarian theory, a material principle of distributive justice, dictates that equitable access to health resources should be afforded to all members of the community [3]. Variations in access to healthcare based on non-clinical factors such as demographic and individual attributes continue to impact safety and quality of care in high income countries [4]. This variation can influence timely access to health resources when errors in reasoning processes missed or delayed diagnosis [5]. Diagnostic related medical errors are common and are a major contributor of patient harm [6]. In Australia, it has been estimated that 140,000 cases of diagnostic error occur annually, leading to 2,000-4,000 deaths [7].

Diagnostic and treatment errors are commonly attributed to cognitive factors [7]. Clinical decision-making, however, is an inherently social activity, and as a result, is subject to a range of situational factors. In this context, health professionals routinely reason their way through a complex array of decisions under conditions of uncertainty [2]. Cognitive and implicit bias are identified as two distinct sub-types influencing decision making in practice [8]. To date, effective strategies to systematically address diagnostic and treatment errors have mainly focussed on addressing the knowledge deficits of health professionals. This has been done with limited reference to curriculum development and pedagogical strategies to prepare the future health workforce. Education programs for new health professionals may provide an opportunity to systematically raise awareness of the role of bias in diagnostic and treatment errors and potentially mitigate the influence of bias on clinical decision making.

### Cognitive bias

Tversky and Kahneman introduced the term ‘cognitive bias’ in the early 1970s to explain people’s systematic, but flawed approach to judgments and decision making [9]. Bias occurs when clinicians incorrectly interpret or apply the clinical data they have obtained [9]. It has been posited that health professionals are susceptible to cognitive biases when making clinical decisions under conditions of uncertainty [10–12]. To date, over 30 cognitive biases that impact medical decision making have been identified, however there may be many more in existence [13]. Common types of cognitive biases include availability, anchoring, confirmatory, and stereotyping biases [14, 15]. Importantly cognitive bias relates to how clinicians perceive and interpret both subjective and objective clinical data. Implicit bias influences how clinicians perceive and respond to others based on personal characteristics, such as sex, age, gender, weight, race, religion, socioeconomic status, and/or bodily difference [8]

Cognitive bias results from major processes that govern human cognition. Tversky and Kahneman’s [16] influential dual process model of decision making posits that humans use two systems to process information. System 1 underlies fast, automatic, intuitive decisions that make incomplete use of available information and rational processes, and instead rely on unconscious use of heuristics, or automatic thought patterns (short cuts) that reduce a complex scenario into a simpler set of parameters to facilitate efficient decision making [1, 16]. In general, System 1 thinking is often a decision making ‘default’ because it is quick, efficient, and less taxing [8]. Because of these features, it could be argued that System 1 thinking is also crucial in responding to emergency situations. While this approach usually does facilitate correct decision making, it is also open to error and therefore is an issue for clinicians and their patients [16]. In contrast, System 2 thinking is characterized by slow, effortful, deliberate decisions, associated with unfamiliar or difficult situations or judgements [16]. However, the more knowledge and experience a clinician acquires, the more mental short cuts they also possess, leading to greater adoption of Systems 1 type thinking [8]. In the healthcare setting, clinical decisions are often made under conditions of stress and/or uncertainty. Therefore, clinicians tend to, and sometimes must, adopt System 1 type thinking and employ heuristics as a cognitive resource saving strategy when making decisions. Notwithstanding this theory, commentators have called into question the view that awareness raising in and of itself reduces the impacts of cognitive bias and suggest that other contextual factors might be at play [17–20].

### Implicit bias

Implicit bias involves the unconscious attitudes that precipitate unintentional discriminatory behaviour [21, 22]. Automatically classifying or grouping patients based on certain characteristics affects clinicians’ judgements relating to, and their interactions with, patients [21, 22]. Implicit bias can disadvantage those that are already vulnerable and impacts all stages of the clinician/patient relationship [23].

For over a decade, commentators have recognized an association between implicit bias and adverse events in hospitals. Instances of implicit bias in healthcare include poor pain management toward Black patients [24], suboptimal management of suicidal ideation in the elderly [25], and delayed diagnosis of chronic obstructive pulmonary disease among women compared to men despite having similar signs and symptoms [26].

How we perceive others, and the development of social or cultural biases, evolves from early childhood experiences [8]. It is thought that we develop these pathways to help provide a quick and efficient determination of groups of people [8]. This may be expressed as overt biases (i.e., explicit) such as open racism or homophobia, or more commonly as implicit bias. Studies have shown that with age, our explicit bias views reduce whereas our implicit bias views remain the same [27]. Healthcare professionals have been shown to manifest implicit biases similar to general population levels [23], which presents a concerning influence on decisions and judgements made by clinicians.

As there is potential for cognitive and implicit biases to unduly influence clinical decisions related to patient assessment (diagnostic and treatment decisions) and management (omissions), strategies to mitigate these known risks are urgently needed. Due to their unconscious nature, biases are inherently fraught and challenging to overcome [21]. Debiasing strategies in clinical medicine have been studied extensively [7, 28], and there is some evidence that targeted training can improve recognition of cognitive biases [29]. To date, little work has been undertaken to identify debiasing strategies in nursing and allied health professions [14, 30]. Yet, despite recognition of the importance of incorporating instructions about cognitive and implicit biases into tertiary level medical and health sciences curricula, the extent to which this occurs, and specific pedagogical techniques and strategies that are used, have not been systematically reported. The primary research question addressed in this review is *What approaches to teaching cognitive and implicit bias, for entry to practice students, have been studied, and what are the evidence gaps that remain?*

Secondary questions include:

- What pedogeological approaches are used when teaching healthcare students about cognitive and implicit bias?
- What educational techniques/tools/strategies are used to deliver educational interventions that attempt to mitigate cognitive and implicit biases?
- Which specific types of cognitive and implicit biases, if any, are being addressed?
- How do educators assess/evaluate the effectiveness of educational interventions designed to mitigate cognitive and/or implicit bias?

For this scoping review, tertiary level education refers to education that, upon successful completion, receives an award spanning the Australian Qualifications Framework (AQF) levels 5-10 [31]. These awards may include bachelor’s degrees; graduate certificates and diplomas; master’s degrees; and higher doctoral degrees [31]. Health disciplines included in this review include medicine, nursing and midwifery, allied health, and biomedicine.

## Method

This review was guided by Joanna Briggs Institute (JBI) methodology for scoping reviews [32] and registered with Open Science Framework registries (https://osf.io/4bpqe). The Population, Concept and Context (PCC) framework was used to guide the purpose of the review and construct the eligibility criteria for papers to be included (see Table 1). The population of interest was pre-registration healthcare-based students undertaking tertiary level education in any healthcare-related discipline – that is, the future workforce. As such, studies focusing health clinicians alone, were excluded as it was considered that practicing clinicians in the current workforce have greater experience in the delivery of care with structural supports in place to mitigate bias. Studies comparing both students and practicing clinicians were excluded if the results were not presented separately for each cohort. Further, studies exploring bias relating to student enrolments at universities were excluded. The concept for this review focused on- research reports exploring pedagogical approaches and/or educational techniques, strategies, and/or teaching tools to reduce the influence of bias in health clinicians' decision making. Studies exploring bias without identifying an educational strategy/approach were excluded. All types of cognitive biases (specified either broadly or specifically) or the terms *‘*cognitive bias*’* or *‘cognitive errors’* and *implicit bias* were included. Papers that referred to *'decision making'* or *‘clinical/diagnostic reasoning’* in general without specifically referring to cognitive/implicit biases were excluded given that many factors and processes besides cognitive/implicit bias are involved in reasoning and decision making. The context of selected studies was settings in which healthcare can be taught, such as universities, hospitals, residential facilities, and clinics. Continuing Professional Development programs, which are undertaken by practicing professionals within health organisations were excluded, given that such courses are not targeted at entry to practice students.

**Table 1:** Inclusion and exclusion criteria

**Inclusion**	**Exclusion**
Population of interest: • Tertiary students in healthcare disciplines Concept • Pedagogical approaches and/or educational techniques, strategies, teaching tools • Implicit bias and cognitive biases (specified clearly), or the term ‘cognitive errors’ Context • Settings where tertiary level healthcare can be taught Other: • Quantitative, Qualitative research reports • English Language • Biases targeted towards patients only	Population of interest: • Health clinicians • University administrators/enrolment officers Concept • Does not use any of the term cognitive terms, implicit bias, or the names of specific biases • Bias in a population without identifying an educational intervention/strategy to address bias Context • CPD (Continuing Professional Development) type courses aimed at practicing professionals Other • Text or commentary/opinion pieces • Protocols

A search strategy was developed to identify published and unpublished quantitative and qualitative studies that presented original data to support their findings. An initial limited search of MEDLINE and Cumulative Index to Nursing & Allied Health (CINAHL) was performed to identify articles on cognitive and implicit bias to identify relevant keywords and index terms to develop the full search strategy. The complete search strategy was then applied to CINAHL, Cochrane, JBI, Medline, ERIC, Embase and PsycINFO databases in May 2022 (last searched conducted 27 May 2022). Grey literature was identified by searching Open Dissertations and Google Scholar. Year limits were not placed on the search. The reference lists of systematic and scoping reviews identified at the full text screening phase were also subject to the screening process. Conference abstracts, protocols, editorials, discussion, and opinion papers were excluded as they were considered to have insufficient information, and/or have the potential to reflect individual preferences or interests. Studies were limited to those published in English and focusing on humans. An example of the search string used for Medline OVID can be found as part of supplementary material.

A data extraction tool (Table 2) was developed by the investigative team to guide data collection relating to population, concept, context, study methods and key findings relevant to this review. To assess inter-rater reliability, two members of the research team independently used the tool to extract data from 10% of the identified articles. Two rounds of testing were required to reach a threshold agreement of 95%. Two independent reviewers then completed title and abstract screening, and full text screening. Any disagreements that arose between reviewers at each stage of the selection process were resolved through discussion with a third member of the investigative team. Once data was extracted from the included articles, both reviewers then analysed each bias separately according to 1) the approach to education and 2) the approach to learning assessment employed by the study.

**Table 2:** Data extraction tool

Publication Details	First author
	Year
	Title
	Country of origin
Study Details	Aim/Purpose of study
	Design
Population	Sample size
	Discipline (medicine, nursing etc.)
	Degree level
Concept	Type of bias
	Pedagogical Practice/Concepts/Techniques (including mode of delivery)
	Method of evaluation of learning
Context	Setting of intervention
	Education provider
Findings/Outcomes	Including any limitations

## Results

**Fig. 1:**
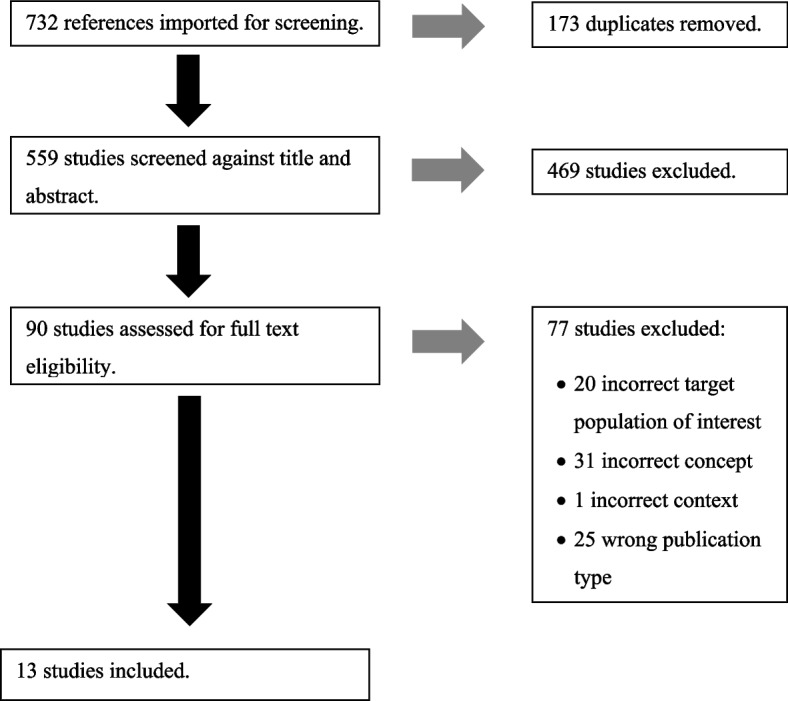
PRISMA ScR flow diagram reporting the search, screening, and study selection process

The search strategy (including studies identified in other reviews) yielded 732 studies. These citations were uploaded into EndNote (Clarivate, version 9.3.3) and 155 duplicates were removed. The remaining citations were then uploaded to Covidence (version 2974 da970e19), and another 18 duplicates were removed. Two independent reviewers examined the titles and abstracts of 559 papers against the inclusion and exclusion criteria (see Table 1), and 90 papers progressed to full text review. At the full text screening phase, agreement could not be reached on two studies, so a third member of the investigative team was approached to independently review these papers for eligibility. Following the full text review, 13 articles were included in the review. Reasons for exclusion of articles at full text are reported in the Preferred Reporting Items for Systematic Reviews and Meta-analyses Extension for Scoping Review (PRISMA-ScR) flow diagram [33] (Fig. 1).

Study characteristics of the 13 papers included in this review are outlined in Table 3. Publication years ranged from 1996 to 2021; the majority (*n=*12) were published within the past 10 years. Most publications originated from the United States (*n=*9), followed by Malaysia (*n=*2) and Canada (*n=*2). Educational practice in medicine accounted for a majority of studies (*n=*8), followed by nursing and midwifery (*n=*2), biomedicine (*n=*2), and pharmacy (*n=*1). Eight studies focused on implicit bias and 5 studies focused on cognitive bias. All studies were presented by a university (*n=*13) and most education occurred in a university setting (*n=*12). Study designs included qualitative studies (*n=*2), randomized controlled trials (*n=*1), mixed methods (*n=*2), quasi-experimental (*n=*4) and cross-sectional (*n=*4).

**Table 3:** Characteristics of studies

**Author/ Year/ Country**	**Aim/Purpose**	**Study Design**	**Sample /** **Degree level / Education provider / Setting**	**Discipline**	**Types of Bias**	**Pedagogical approach/ Educational techniques/tools/strategies**	**Approach to Assessment**	**Outcomes Assessed**	**Results**
**COGNITIVE BIAS**									
Chew et al. [34] (2016) Malaysia	To test the hypothesis that the TWED checklist facilitates metacognition among medical students so that they can make better-quality clinical decisions.	Quasi-experimental	40 students (intervention: 21; control: 19) Bachelor's/ Undergraduate University provider and setting	Medicine	Cognitive bias: - Anchoring bias - Availability bias - Confirmation bias - Search satisficing	1 x 90-minute tutorial on dual process thinking, cognitive biases, debiasing strategies and the TWED checklist (T = threat, W = what else, E = evidence and D = dispositional factors)	20 true/false question quiz based on 5 x clinical case scenarios	The ability of students to generate a second more serious diagnosis, and their ability to decide on appropriate investigations and treatment plans	Overall mean scores in all 5 clinical cases Intervention group: 18.50 +/- 4.45 marks Control group: 12.50 +/- 2.84marks *p*<0.001
Chew et al. [35] (2017) Malaysia	To test the hypothesis that the application of a mnemonic checklist aids the script evaluation stage by stimulating the consideration of additional patient data, thus leading to better clinical decisions.	Quasi-experimental	88 final year students (intervention: 48; control: 40) Bachelor's/Undergraduate. University provider and setting	Medicine	Cognitive bias: - Premature closure	2-hour tutorial covering topics of factors that contribute to diagnostic errors and strategies to minimize them using case-based discussions. The TWED checklist (Threat, what else, Evidence, Dispositional influences) was also introduced and explained to students.	Script concordance test consisting of 10 cases with 3 items	Participant’s decisions on the likelihood of a given hypothesis when additional patient data is provided.	The median total scores for the intervention group and the control group were: 18.65 (IQR 16.96 - 20.34) and 18.15(IQR 16.79 - 19.37) out of 30, respectively. *U*=792 *z*=−1.408 *p*=0.159.
Deveau et al. [36] (2021) Canada	To discuss a pilot project using simulation-based learning to integrate exploration of thinking and identification of cognitive biases	Cross sectional	19 students Bachelor's/Undergraduate University provider and setting	Nursing/ Midwifery	Cognitive bias: - Anchoring bias - Availability bias - Confirmation bias - Framing effect - Search satisficing - Premature closure	Simulation based learning. Simulation (case scenario - 21 year old single mother with her infant son)	Debriefing post simulation (using good judgement framework and a cognitive autopsy approach)	Exploration of clinical reasoning and identification of cognitive biases	Clinical reasoning: 42% (*n=*8) identified the correct clinical impression. Identification of cognitive biases: Anchoring bias 63% (*n=*12) Confirmation bias 47% (*n=*9) Search satisficing 42% (*n=*8) Unpacking principle 42% (*n=*8) Premature closure 32% (*n=*6) Availability bias 21% (*n=*4) Overconfidence 21% (*n=*4) Framing 16% (*n=*3) Diagnostic momentum 16% (*n=*3)
Hershberger et al. [37] (1996) USA	To determine: (1) how capable medical students and practicing physicians are in avoiding cognitive biases in medical decision-making, (2) whether susceptibility to cognitive bias varies by medical specialty, and (3) whether awareness of cognitive bias in medical decision-making can be taught to medical students.	Quasi-experimental	230 students (118 intervention group; 112 control group) Bachelor's/ Undergraduate University provider and setting	Medicine	Cognitive bias: - Availability bias - Confirmation bias - Representative-ness heuristic	1 x Seminar to address predictable tendencies in information processing that can adversely impact decision making	The Inventory of Cognitive Biases in Medicine (ICBM) was used to determine the influence of bias, and the effectiveness of the seminar in teaching the principles of cognitive bias in medical decision-making.	1: The extent of cognitive bias in medical decision-making and whether experienced physicians differed from novices 2: If awareness of cognitive bias in medical decision-making could be taught.	1: Preclinical medical students' mean score: 40.7% (SD = 12.5%). Practicing physicians mean score: 49.0% (SD = 14.9%). Influence of bias (49% vs 41%, t = 4.07, *p <* 0.001). 2: Intervention group: 40.7% (SD = 12.5%). Control group: 55% (no SD provided) t = 7.83, *p <* 0.001
Sherbino et al. [18] (2014) Canada	To determine the effect of cognitive forcing strategies (CFS) training on diagnostic error in senior medical students	Quasi-experimental	198 final year students of a 3-year curriculum (intervention: 145, control: 46) Bachelor/Under-graduate University provider Hospital/Clinic setting	Medicine	Cognitive bias: - Availability bias - Search satisficing	1 x 90 minute interactive case-based seminar facilitated by expert EM clinicians experienced with teaching Cognitive Forcing Strategies based on Croskerry’s model.	A 2-hour, 48 question, computer-based examination using case studies	Search Satisficing Bias: Compared the proportion of students in the intervention group and the control group who searched for a second diagnosis and the proportion whose second diagnosis was correct, and the proportion that identified a second diagnosis was compared for false positive cases (for which there was no second diagnosis). Availability Bias: Compared the proportion that identified the uncommon diagnosis.	Search Satisficing Bias: 52% of intervention group and 48% in the control group initiated a search for the second diagnosis (x^2^= 0.13, df = 1, p = 0.91). Of these students, 54% of the control group correctly identified the second diagnosis compared to 48% in the control group (x^2^ = 2.25, df = 1, p = 0.13). Second diagnosis/false positive cases, 64% of the intervention group and 77% of the control group incorrectly identified it (x2 = 2.38, df = 1, p = 0.12). Availability Bias: 45% in each group identified the uncommon correct diagnosis (x^2^ = 0.001, df = 1, p = 0.98).
**IMPLICIT BIAS**									
Avant et al. [38] (2019) USA	To evaluate an elective course focused on exposing students to the root causes of health disparities, con-temporary factors that perpetuate disparities, and evidence-based policies to reduce health disparities.	Qualitative	9 second- and third-year students Undergraduate/ Bachelor’s University provider and setting	Pharmacy	Implicit bias: - Race	Course on bias and structural inequalities as drivers of inequality. Students learned through self-reflection; perspective taking; and group activities. Active learning used throughout the course to promote self-discovery (e.g., social identity mapping).	- Short answer exam - learning management system discussion threads - weekly reflections - photo presentation on social determinants of health and equity, - comparing two racially segregated neighboring suburbs	Identified 5 strategies to facilitate this course: Knowledge and understanding of drivers of health disparities, bias and structural inequalities. Regarding racial/ethnic health disparities; and encouraging personal awareness of privileged and marginalized identities.	Five themes emerged from student reflections underlying strategies to facilitate this course: create and maintain a welcoming and inclusive learning environment. (2) utilize experiential learning for personal awareness development and knowledge expansion. incorporate intergroup diversity to empower learners to create change. anticipate and acknowledge emotions to facilitate learning and: 5) provide students with an opportunity to complete a final self-reflection paper.
Gatewood et al. [39] (2019) USA	To raise awareness of implicit bias and its influence on health outcomes and support a discussion on ways to mitigate the impact of implicit bias.	Cross sectional	110 nursing students from 4 universities Bachelor's/ Undergraduate 13 students); Masters (33 students); Doctorate (64 students) University provider and setting	Nursing/ Midwifery	Implicit bias: - Race	3-part educational activity: 1)preparatory activities (video and articles on implicit bias in healthcare and the IAT). 2)IAT. 3)Discussion activity regarding results of IAT and potential actions to mitigate effects of implicit bias.	- Facilitated discussion about activity - Online evaluation of usefulness of the assignment	Students level of agreement preparatory material provide to increase awareness of the effects of implicit bias (IB) on quality in healthcare, identify a resource for self-assessment of IB using the IAT, and integrate knowledge of students' own IBs into their nursing care to improve the quality of their care.	Preparatory learning activities were helpful: Video: 89% strongly agree or agree Readings: 86% strongly agree or agree. Increased awareness of personal biases: 83% strongly agree or agree. Recognition of their IB (via the IAT) would be helpful in managing their nursing care: 90% strongly agree or agree. Usefulness of discussion and overall impression of activity to manage IB: Discussion - 70% strongly agree or agree. Overall - 69% strongly agree and agree.
Gonzalez et al. [40] (2014) USA	To describe an educational intervention addressing both health disparities and physician implicit bias and the results of a subsequent survey exploring medical students’ attitudes and beliefs toward subconscious bias and health disparities	Cross sectional	218 3^rd^-year students over a 2-year period (2 cohorts) Bachelor's/ Undergraduate University provider and setting	Medicine	Implicit bias: - Race - Religion	Single session within a course. Preparatory activities: pre-reading on health disparities, physician behaviour and implicit bias; written reflection; IAT Session: Faculty-led discussion on health disparities; students’ personal experiences; and effect of bias on decisions.	- A 15-question anonymous 4-point Likert scale survey	Students’ attitudes and beliefs toward subconscious bias and health disparities and to evaluate the association between students’ self-assessment and what they believed or observed about health disparities following an educational intervention and completion of the IAT.	Survey response rate: 69%. 22% of responders were “strongly disagreed or disagreed (deniers) to the statement Unconscious bias might affect some of my clinical decisions or behaviors compared to 77% of responders who strongly agreed or agreed (acceptors) with the statement. 9% of deniers and 1% of acceptors disagreed with the statement Health -Disparities Do Not Exist in the United States (p = 0.02)
Gonzalez et al. [41] (2020) USA	To provide an overview of a skills based elective course and its evaluation in implicit bias recognition and management	Mixed method	First year students over 3 years (3 cohorts) Bachelor's/ Undergraduate University provider and setting	Medicine	Implicit bias: Not specified	Elective subject consisting of 9 x 1.5hour sessions. The conceptual framework developed by Teal et al. was used to guide instructional design. The course consisted of 2 sections. Section 1: reflections on own biases and experience of completing the IAT; perspective-taking exercises. Section 2: roleplays and brainstorming session on addressing perceived bias during witnessed encounters.	2 x Focus groups during and post-completion of course, exploring perceptions of course.	The student’s ability to: Recognize when implicit bias may influence one’s own communication with a patient or peer. Advocate on behalf of patients when perceiving bias in a witnessed encounter; and 3. Address biased comments made within the learning environment.	3 themes emerged from analysis of focus groups: 1: Student engagement can be enhanced. 2: Instruction is empowering, and 3: It (addressing bias in one’s own and witnessed encounters) can be done!
Matharu et al. [42] (2014) USA	To determine whether an innovative educational intervention (reading a play about obesity) could diminish obesity prejudice relative to a standard medical lecture	Randomized controlled trial	129 1st-4th year students from four universities Bachelor's/ Undergraduate University provider and setting	Medicine	Implicit bias: - Weight bias	Intervention: 1 hour dramatic reading of a script incorporating narratives about their weight in the context of social discrimination. Control group: 1 hour lecture on the medical management of obesity.	- The obesity specific IAT - The anti-fat attitudes questionnaire. - Jefferson Scale of Physician Empathy (JSPE) - Two open-ended questions: - A) Discuss whether they viewed obesity as a civil rights issue or a medical/public health issue. - B) Formulate a treatment plan for an overweight but otherwise healthy older woman.	Explicit attitudes and implicit bias toward obese individuals were assessed prior to intervention and after four months.	Explicit fat bias difference change in post measures for the intervention group -4.5 +/- 11.5 (P = 0.002), compared to the control group with 0.76 +/- 12.0 (P = 0.61), representing a difference in change from baseline of -5.5 (P = 0.05). Implicit fat bias difference change in post measures for the intervention group -0.03 +/- 0.44, compared to the control group with 0 +/- 0.48 representing a difference in change from baseline of -0.04. Empathy score differences change in post measures for the intervention group -4.7 +/- 13.4 (P = 0.007), compared to the control group with 2 +/- 9.3 (P = 0.02), representing a difference in change from baseline of +2.2. Students in the control group were more likely to endorse a prescriptive model of patient care (P = 0.03).
Motzkus et al. [43] (2019) USA	To explore whether three-hours of focused content and discussion regarding bias and stereotypes could produce student reflections demonstrating insight into these issues.	Qualitative study using grounded theory methodology	250 1^st^-2^nd^ year students Bachelor's/ Undergraduate and Doctorate University setting and provider	Medicine	Implicit bias: Not specified	1 x 3 hour lecture / discussion session on implicit bias and completion of IAT on any topic of students choosing	Written reflective essay	Understanding students’ readiness to accept their implicit biases and to address unconscious bias.	25 themes were identified that could be categorized into 3 areas: 1: Experiencing taking IAT. 2: Bias in medicine; and 3: Prescriptive comments. 84% of students acknowledged the importance of recognizing implicit bias. 60% of students noted that bias affects clinical decision-making. 19% stated they believe it is the physician’s responsibility for dismantling bias. 56% of students felt that the IAT promoted self-reflection. 56% acknowledged that bias is a product of society/culture/ upbringing.
Schwartz et al. [44] (2020) USA	To investigate whether increases in students’ reflective capacity are associated with improved cognitive empathy scores; and whether an increased emphasis on understanding the nature of implicit bias in a medical humanities course, and the resultant pedagogy of discomfort, would cause students to accept the possibility that unconscious bias might affect some of their clinical decisions or behaviors as healthcare professionals.	Mixed methods	34 prospective medical students Masters University setting and provider	Biomedicine	Implicit bias: Not specified	A humanities course designed to promote development of personal and professional skills and identities through readings, quizzes, discussions about communication and implicit bias, and written reflections on service to the community	- Reflective Practice Questionnaire - The Jefferson Scale of Empathy. - Custom designed survey of student’s attitudes toward team service-learning projects and unconscious bias post course completion - Team minutes and reflections	If performing service-learning projects would foster students’ compassion by raising their reflective capacity, empathy, and unconscious bias mitigation.	An increase in reflective capacity scores of prospective medical (MSBS) students in association with written reflections on service learning between August and December 2019 (r=0.26, p=0.02). An increase in the cognitive empathy (JSE) scores of prospective medical (MSBS) students occurred in association with the rise in their reflective capacity (RC) scores between August and December 2019 (r=0.27, p=0.015). Prejudices expressed by students in a survey (and number of times expressed) regarding the biases of which they became aware in their team service-learning experiences: - Age (10) - Homeless people (7) - Culture/Race (6) - Socioeconomic status (4) - Obesity (3) - Gender (3) - Hygiene (1) - Mental health patients (1) - Men I do not know (1) - Environmental (1)
Van Winkle et al. [45] (2021). USA	To determine if remote learning (compared to in class learning) diminished students’ capacity for reflective capacity and cognitive empathy to foster positive attitudes and behaviors for critical reflection, implicit bias mitigation, empathy, and compassion.	Cross sectional	61 prospective students compared to 81 students’ results from three previous cohorts Masters University setting and provider	Biomedicine	Implicit bias: - Age bias - Racial bias - Sex/sex-role bias - Socio-economic status bias - Weight bias. - Substance abuse bias - Disability bias - Mental health bias	Readings, IAT. Classes held by Zoom, including quizzes and application exercises concerning communication skills and implicit bias. Service-learning projects. Team-based activities to write reflections, minutes from team meetings.	- Written assignments. - Reflective Practice Questionnaire - Jefferson Scale of Empathy. - Online survey about opinions concerning implicit bias, team-based learning, and service to the community	Students’ capacity for reflective capacity, empathy, and unconscious bias mitigation.	Reflective capacity scores increased during Medical Humanities course between August and December 2020 (r=0.56, p=0.000). Cognitive empathy (JSE) scores increased during Medical Humanities course between August and December 2020 (r=0.37, p=0.003). Prejudices expressed by students in a survey (and number of times expressed) regarding the biases of which they became aware in their team service-learning experiences: - Ageism (10) - Economic class/homelessness (8) - Obesity (7) - Sexual orientation (7) - Race (3) - Substance abuse/addiction (3) - Strong political opinions (3) - Men (3) - None/no interaction (3) - Disabled (2) - Mental health issues (2) - Veterans (2) - Favor same as me (2) - Appearance/dress (1) - Smokers (1) - Women (1) - Favor CNAs (1) - End of life care (1)

### Cognitive bias and approach to education

Table 4 outlines the categories identified regarding the pedagogical approaches and teaching strategies and techniques used for teaching cognitive biases. Availability bias was the most common cognitive bias covered (*n=*4), followed by confirmation bias (*n=*3) and self-satisficing (*n=*3). The least common biases to be explored were the framing effect (*n=*1) and the representative heuristic (*n=*1). Most studies did not provide a guiding educational philosophy or framework (*n=*4). Sherbino and colleagues [18] used Croskerry’s model to guide their teaching of cognitive forcing strategies. The most common delivery mode was face-to-face teaching (*n=*4). The only other form of delivery of content was through simulation (*n=*1). All papers (*n=*5) focused on a single education session. Four techniques and strategies were identified to teach cognitive biases. These include case-based learning (*n=*2); use of mnemonics (*n=*2); debiasing techniques - not clearly stated (*n=*2); and clinical placement (*n=*1).

**Table 4:** Cognitive bias - approach to education

**Type of Cognitive bias**	**Guiding philosophy/ conceptual framework**	**Educational delivery**	**Number of sessions**	**Techniques/Tools used**
**Anchoring bias** *(n=2 papers)*	• Not clearly stated[34, 36]	• Face to face (lecture/ tutorial)[34, 36] • Simulation[36]	• Single session[34, 36]	• Case-based learning[36] • Mnemonics[34] • Debiasing technique (not clearly stated)[34]
**Availability bias** *(n=4 papers)*	• Croskerry’s model for teaching Cognitive Forcing Strategies[18] • Not clearly stated[34, 36, 37]	• Face to face (lecture/ tutorial)[18, 34, 37] • Simulation[36]	Single session• [18, 34, 36, 37]	• Case-based learning[36, 37] • Clinical placement[18] • Mnemonics[34] • Debiasing technique (not clearly stated)[18, 34]
**Confirmation bias** *(n=3 papers)*	• Not clearly stated[34, 36, 37]	• Face to face (lecture/ tutorial)[34, 37] • Simulation[36]	• Single session[34, 36, 37]	• Case-based learning[36, 37] • Mnemonics[34] • Debiasing technique (not clearly stated)[34]
**Framing effect** *(n=1 paper)*	• Not clearly stated[36]	• Simulation[36]	• Single session[36]	• Case-based learning[36]
**Premature closure** *(n=2 papers)*	• Not clearly stated[35, 36]	• Face to face (lecture/ tutorial)[35] • Simulation[36]	• Single session[35, 36]	• Case-based learning[36] • Mnemonics[35]
**Representativeness heuristic** *(n=1 paper)*	• Not clearly stated[37]	• Face to face (lecture/ tutorial)[37]	• Single session[37]	• Case-based learning[37]
**Self-satisficing** *(n=3 papers)*	• Croskerry’s model for teaching Cognitive Forcing Strategies[18] • Not clearly stated[34, 36]	• Face to face (lecture/ tutorial)[18, 34, 36]	• Single session[18, 34, 36]	• Case-based learning[36] • Mnemonics[34] • Clinical placement[18] • Debiasing technique (not clearly stated)[18, 34]

### Approach to assessment

Table 5 outlines the key themes identified relating to assessment and evaluation of learning of cognitive biases. Three types of assessment were identified from the studies. These were reflective practice (*n=*1), case-based short answer quiz (*n=*2), and case-based multiple choice question quiz (*n=*2).

**Table 5:** Cognitive bias – approach to assessment

**Cognitive bias/Number of studies**	**Reflection**	**Short-answer (case-based)**	**MCQ** **(case-based)**
**Anchoring bias** (*n=2)*	[36]	[34]	
**Availability Bias** *(n=4)*	[36]	[18, 34]	[37]
**Confirmation bias** *(n=3)*	[36]	[34]	[37]
**Framing effect** *(n=1)*	[36]		
**Premature closure** *(n=2)*	[36]	[35]	
**Representativeness heuristic** *(n=1)*			[37]
**Search satisficing** *(n=3)*	[36]	[18, 34]	[35]

*Legend*: *MCQ*   Multiple Choice Question, *ICBM*  Inventory of Cognitive Biases in Medicine

### Implicit bias and approach to education

Table 6 summarizes the categories identified describing pedagogical approaches, teaching strategies and techniques used to address implicit biases. Racial implicit bias was the most common focus within the included studies (*n=*4), followed by implicit bias in general (*n=*3) and weight (obesity) bias (*n=*2). Approaches to teaching other types of implicit bias were identified in one article. The majority of studies (*n=*6) did not indicate a guiding philosophy or conceptual framework to educate students. Half the studies (*n=*4) focused on a single educational session. The most common delivery method was face-to-face (*n=*5), followed by flipped classroom approach (*n=*2), and remote online learning (*n=*1). A wide range of techniques were used to deliver educational content. These included group work (*n=*6); readings (*n=*5); reflection for learning (*n=*5); use of the Implicit Association Test (IAT) [46] (*n=*4); use of media (*n=*2); role play exercises (*n=*1); brainstorming exercises (*n=*1); community service (*n=*1); social identity mapping (*n=*1); photovoice (*n=*1); and the fishbowl technique (*n=*1).

**Table 6:** Implicit bias – approach to education

**Type of Implicit bias/Number of studies**	**Guiding philosophy/ conceptual framework**	**Educational delivery**	**Number of sessions**	**Techniques/Tools used**
Not specified *(n=3)*	• Teal et al. conceptual framework[41] • Not clearly stated[43, 44]	• Face to face[41, 43, 44]	• Single session[43] • Multiple sessions[41, 44]	• Implicit association test (IAT)[43] • Community service[44] • Use of media (videos/recordings)[41] • Role play exercises[41] • Brainstorming exercises[41] • Group work[41, 44] • Readings[44] • Reflection[41, 43, 44]
Age bias *(n=1)*	• Not clearly stated[45]	• Remote/Online[45]	• Multiple sessions[45]	• Implicit association test (IAT)[45] • Readings[45] • Group work[45] • Reflection[45]
Racial bias *(n=4)*	• Active learning/Experiential learning[38] • Not clearly stated[39, 40, 45]	• Face to face[38] • Remote/Online[45] • Flipped classroom[39, 40]	• Single session[39, 40] • Multiple sessions[38, 45]	• Implicit association test (IAT)[39, 40, 45] • Readings[38–40, 45] • Use of media (videos/recordings)[39] • Group work[38–40, 45] • Reflection[38, 45] • Social identity mapping[38] • Photovoice[38] • Fishbowl technique[38]
Sex/Sex role bias *(n=1)*	Not clearly stated[45]	Remote/Online[45]	Multiple sessions[45]	• Implicit association test (IAT)[45] • Readings[45] • Group work[45] • Reflection[44]
Socio-economic status bias *(n=1)*	Not clearly stated[45]	Remote/Online[45]	Multiple sessions[45]	• Implicit association test (IAT)[45] • Readings[45] • Group work[45] • Reflection[45]
Weight bias *(n=2)*	• Not clearly stated[42, 45]	• Face to face[42] • Remote/Online[45]	• Single session[42] • Multiple sessions[45]	• Case-based learning[42] • Implicit association test (IAT)[45] • Dramatic reading of a play[42] • Readings[45] • Group work[45] • Reflections[45]
Substance abuse bias *(n=1)*	• Not clearly stated[45]	• Remote/Online[45]	• Multiple sessions[45]	• Implicit association test (IAT)[45] • Readings[45] • Group Work[45] • Reflection[45]
Disability bias *(n=1)*	• Not clearly stated[45]	• Remote/Online[45]	• Multiple sessions[45]	• Implicit association test (IAT)[45] • Readings[45] • Group work[45] • Reflection[45]
Mental health bias *(n=1)*	• Not clearly stated[45]	• Remote/ Online[45]	• Multiple sessions[45]	• Implicit association test (IAT)[45] • Readings[45] • Group work[45] • Reflection[45]
Religion bias *(n=1)*	• Not clearly stated[40]	• Flipped classroom[40]	• Single session[40]	• Implicit association test (IAT)[40] • Readings[40] • Group Discussion[40]

### Approach to assessment

Table 7 provides an overview of the assessment and evaluation of learning strategies employed to address specific types of implicit biases. A wide range of assessment items were identified. The most common assessment tools were the use of a written reflective essay (*n=*3) and the Jefferson Scale of Empathy standardized assessment tool (*n=*3) [47]. Other assessment strategies included the following: a survey of the student’s perception of the course (*n=*2); the reflective practice questionnaire standardized tool (*n=*2); role play of skills assessment (*n=*1), portfolio of work (*n=*1); short answer exam (*n=*1); discussion threads (*n=*1); class participation (*n=*1); and the Anti-fat Attitudes Questionnaire standardized tool (*n=*1).

**Table 7:** Implicit bias – approach to assessment

**Type of implicit bias**	**Roleplay skills assessment**	**Written reflective essay**	**Portfolio (photo essay)**	**Short-answer exam**	**LMS discussion threads**	**Participation in class**	**Survey about perceptions of the course**	**Standardised assessments**		
								**Reflective practice questionnaire**	**The Jefferson Scale of Empathy**	**Anti-fat Attitudes questionnaire**
**Not specified** ***(n=3 papers)***	[41]	[43]						[44]	[44]	
**Age bias** ***(n=1 paper)***		[45]					[45]	[45]	[45]	
**Racial bias** **(*n=*4 papers)**		[39, 45]	[38]	[38]	[38]	[38]	[40, 45]	[45]	[45]	
**Sex/Sex role bias** ***(n=1 paper)***		[45]					[45]	[45]	[45]	
**Socio-economic status bias** ***(n=1 paper)***		[45]					[45]	[45]	[45]	
**Weight bias** ***(n=2 papers)***		[45]					[45]	[45]	[42, 45]	[42]
**Substance abuse bias** ***(n=1 paper)***		[45]					[45]	[45]	[45]	
**Disability bias** ***(n=1 paper)***		[45]					[45]	[45]	[45]	
**Mental Health bias** ***(n=1 paper)***		[45]					[45]	[45]	[45]	
**Religion** ***(n=1 paper)***							[40]			

## Discussion

In this review we sought to answer the primary question: *what approaches to teaching cognitive and implicit bias, for entry to practice students, have been studied and what are the evidence gaps that remain?* Our scope identified a small body of published literature describing the phenomena of cognitive and/or implicit bias and its application in curricula for courses leading to registration in the health professions. Most studies in the current review described teaching sessions delivered to medical students undertaking university-based programs in North America, where the focus was addressing implicit bias, as opposed to cognitive bias.

This review highlights a critical gap in the evidence available outlining how educators of health professionals teach cognitive and implicit bias and their impact on diagnostic and treatment-based decisions. This gap is notable for two reasons. First, it is well-recognised that bias in healthcare remains systemic and has potentially devastating impacts on safety and quality of care [48, 49]. Second, the responsibilities now incumbent on employers of health professionals in many jurisdictions to meet their obligations under anti-discrimination law mean that attention is paid to educating the workforce about implicit bias and strategies needed to address it. In this respect, tertiary education providers must work proactively to develop evidence-based approaches to learning and teaching aimed at mitigating all forms of bias that have the potential to impact the delivery of high-quality healthcare.

### Cognitive bias

In addressing the potential influence of heuristic thinking on diagnostic and therapeutic decision-making, availability bias – the tendency to use information that comes to mind quickly when making judgments – was the focus of most of the strategies described [50]. This finding aligns with the view that the availability heuristic is among the most utilized by medical practitioners when making diagnostic decisions in practice [50]. A recent experimental study of medical residents’ diagnostic reasoning for cases of dengue fever by Li and colleagues [50] found that availability bias led to diagnostic error and that misdiagnosis cannot always be effectively addressed using a reflective approach. Other heuristics specifically identified in our scoping review included self-satisficing - searching through available diagnostic alternatives until an acceptable threshold is met [51] - and confirmation bias - the tendency to search for, interpret, favor, and recall information in a way that confirms or supports one's prior beliefs or values [51]. Less frequently explored were the framing effect – the same problem is presented in multiple settings, but different representations of information influence the outcome [52] —and the representative heuristic — knowledge of prior probabilities of a characteristic in a similar population incorrectly influence decision outcome [53].

In terms of theoretical orientation taken to explore cognitive bias in educational programs, most of these studies drew, to some extent, on dual systems theory [16]. Sherbino and colleagues [18] adopted Croskerry’s model to evaluate the effect of teaching of cognitive forcing strategies on diagnostic error in medical students. Croskerry’s model proposes that a prerequisite to addressing the problem of cognitive error (in emergency medicine) is to first ensure learners understand dual processing theory. While Croskerry recommended strategies to deal with different categories of error, along with an awareness of how cognitive biases can influence patient outcomes in different clinical situations as a strategy, Sherbino and colleagues [18], found this conceptual framework to be ineffective, which is a notion that has gathered support recently [17, 19, 20].

### Implicit bias

In the 8 studies addressing implicit bias, race, weight (obesity), age, disability and substance use, and mental illness were the attributes addressed using a range of educational approaches. While much of the literature was published in North America, all implicit biases noted may be considered protected attributes, and as such, characteristics against which it is unlawful to discriminate [54]. Unlike the studies focused on cognitive bias, none of the studies exploring implicit bias cited specific educational theories to inform pedagogy and most utilized a single session to address the issue via face-to-face delivery. Considering both the ethical responsibilities outlined in health professionals’ codes of conduct and the legislative frameworks in place in many jurisdictions to protect citizens against discrimination, it is timely to consider how a curriculum to address implicit bias based on the different types of protected attributes might be beneficial to inform programs educating the future health workforce.

A variety of techniques were identified to engage students in and reflect on learning about implicit bias. These included working in groups, role play, fishbowl technique and brainstorming. Innovative participatory methods were also reported in a small number of studies to engage students to reflect on their own identity such as social identity mapping and the use of photovoice. Several studies reported using the Implicit Association Test (IAT) as a starting point for critical reflection, which is consistent with a review by Kruse and colleagues [55], who found that the IAT is commonly incorporated into education for healthcare students and provides a strategy to assess awareness of implicit biases. Few of the included studies employed strategies in practical or real-world environments. That is, in contrast to reviews of interventions to study or mitigate biases in healthcare professionals [56, 57], only one of the included studies in this review referred to service learning or patient/social contact as pedagogical strategies, despite evidence that such learning experiences can lead to bias mitigation by increasing compassion and reflective capacity [58].

Implicit bias by its very nature is unconscious, meaning the actions and decisions of health professionals are influenced without their awareness [59]. However, none of the included papers explored the concept of *Speaking up* for patient safety. *Speaking up* refers to health professionals expressing concerns if they observe the actions of others (e.g. mistakes, lapses, rule breaking) that can negatively impact patient safety and quality of care [60]. Barriers to staff speaking up are well known and include institutional, interpersonal, and individual factors [61]. Educating tertiary students regarding their knowledge and awareness of implicit bias should be accompanied with a framework that provides them with the tools and knowledge to *speak up* if they were to observe bias in action.

The findings from this review indicate that assessment of student learning about the nature and impact of implicit bias has tended to rely on traditional approaches such as tests, written reflective essays and exams. Some self-assessment tools such as the Jefferson Scale of Empathy standardized assessment and the Anti-fat Attitudes Questionnaire were employed to evaluate learning. Less commonly authentic modes of assessment such as portfolio work were utilized to assess learner knowledge.

The complex and diverse set of competencies that are required of health professionals means that no single approach to assessment is adequate [62]. In terms of Miller’s pyramid, the predominance of written, test-based assessments employed in the current review indicates that bias mitigation interventions in entry-to-practice degrees tend to evaluate student learning at the lowest levels of ‘Knows’ and/or ‘Knows How.’ Assessing the higher levels of Miller’s pyramid – particularly the ‘Does’ level – requires assessing students in real-world settings such as a clinical context [63].

The importance of multiple and varied approaches to student assessment is highlighted in Sukhera and Watling’s [64] framework for incorporating recognition of implicit bias into education for health professionals. The framework proposes that comprehensively assessing learning in this area requires several different assessment strategies targeting distinct aspects of implicit bias recognition. For example, whereas tests assess knowledge about implicit bias, reflective exercises and portfolios are more appropriate for assessing students’ development of self-awareness of their own implicit biases, while observed clinical evaluations or assessments of students during practicums or other real-world settings, are appropriate for assessing the development of conscious efforts to overcome implicit bias [64]. Considering this recommendation, it is notable that few included studies used numerous and/or diverse assessment methods. Furthermore, none of the included studies employed the observation of clinical evaluations, suggesting there is limited assessment of the extent to which students develop and maintain conscious efforts to overcome biases. This finding is surprising, given it is well recognized that clinical placements are an essential component of clinical education [65, 66].

### Limitations

While the search strategy included eight databases and Google, not all relevant papers may have been identified. Similarly, limiting our search strategy to English publications may have excluded relevant papers. Our population of interest was tertiary students in healthcare disciplines, and as a consequence, our exclusion of studies with mixed samples of students and healthcare professionals, and students and residents, may have potentially omitted studies that employed pedagogical and/or assessment strategies not otherwise identified here. Papers that were excluded due to incorrect population, concept or context during screening can be found as part of supplementary material. As a scoping review, our study did not include quality appraisal or grading of evidence. Nonetheless it should be noted that the high degree of variability in methods and outcomes limits more rigorous appraisal of the evidence.

### Implications for teaching and learning

Antidiscrimination laws in many countries now rule it unlawful to delay or limit access to health care based on specified personal characteristics, including but not limited to age, disability, race, sex or gender identity [67, 68]. As an example, within Australia, federal laws of this type include the Age Discrimination Act 2004 [69], the Disability Discrimination Act 1992 [70], Racial Discrimination Act 1975 [71] and the Sex Discrimination Act 1984 [72]. Understanding that these laws apply to cases of explicit and overt discrimination, it is unsure if they could be enforced if implicit bias was found to be a contributing factor in a coronal inquiry into the death of an individual. Given the potential impacts of bias due to discrimination on safe, timely access to health care, it is incumbent upon tertiary education providers responsible for training our future health workforce to ensure graduates receive education of the nature and type of clinical errors or practice differences that may result from implicit bias and the strategies to mitigate these.

Training in the context of direct participation in clinical care (during a clinical placement) plays a major role in health professional education and preparedness [65, 66], therefore educators need to design learning objectives for placements that focus on translating knowledge and awareness of bias into practice and the leadership to respond when they observe the actions of others for the benefit of patient care and safety.

## Conclusion

In this review, we sought to explore what approaches to teaching cognitive and implicit bias have been studied and what are the evidence gaps that remain for pre-registration students. A range of pedagogical strategies were employed; most commonly, these were face-to-face, class-based activities such as lectures, tutorials, and simulations, and were delivered predominantly across one as opposed to multiple sessions. Assessments of student learning were primarily based on tests and personal reflection. There was limited use of real-world settings (i.e., placements or practicums) to educate students about or build skills in biases and their mitigation, and no studies assessed students’ learning in practical settings. Further work is urgently required to develop innovative pedagogical approaches to developing the skills of future healthcare professions in recognising and mitigating the effect of different biases, and approaches to evaluate these skills comprehensively and meaningfully. There may be a valuable opportunity in exploring approaches to building these skills in the real-world settings that will be the workplaces of our future healthcare workers.

## Supplementary Information

**Additional file 1. **Search Strategy used for OVID Medline.

**Additional file 2:** **Supplementary Table 1.** Exclude Articles.

## Data Availability

All data generated or analysed during this study are included in this published article and its supplementary information files.
