# Supplementary material for: Educational strategies in the health professions to mitigate cognitive and implicit bias impact on decision making: a scoping review
Source: BMC Med Educ. 2023 Jun 20;23:455. doi: 10.1186/s12909-023-04371-5 (PMC10280953; doi:10.1186/s12909-023-04371-5)
Supplement: Supplementary file 1 — Additional file 1. Search Strategy used for OVID Medline. [file 12909_2023_4371_MOESM1_ESM.docx]

Search Strategy OVID Medline May 11, 2022

1 student*.mp.

2 medicine.mp.

3 medical.mp.

4 nurs*.mp.

5 midwi*.mp.

6 Allied health.mp.

7 psycholog*.mp.

8 dentis*.mp.

9 social work*.mp.

10 optom*.mp.

11 opthal*.mp.

12 physiotherap*.mp.

13 physical therap*.mp.

14 speech therap*.mp.

15 audiolo*.mp.

16 audiolo*.mp.

17 audiometr*.mp.

18 speech patholog*.mp.

19 radiolog*.mp.

20 radiogr*.mp.

21 radiogr*.mp.

22 occupational therap*.mp.

23 health profession*.mp.

24 2 or 3 or 4 or 5 or 6 or 7 or 8 or 9 or 10 or 11 or 12 or 13 or 14 or 15 or 16 or 17 or 18 or 19 or 20 or 21 or 22 or 23

25 1 and 24

26 bias*.mp.

27 cognitive bias*.mp.

28 implicit bias*.mp.

29 debias*.mp.

30 availability bias*.mp.

31 anchoring bias*.mp.

32 confirmation bias*.mp.

33 racial bias*.mp.

34 gender bias*.mp.

35 sex bias*.mp.

36 sex role bias*.mp.

37 socioeconomic status bias*.mp.

38 age bias*.mp.

39 dual process.mp.

40 cognitive error.mp.

41 fundamental attribution error.mp.

42 representative* heuristic.mp.

43 heuristic*.mp.

44 optimistic bias*.mp.

45 hindsight bias*.mp.

46 omission bias*.mp.

47 base rate neglect.mp.

48 bandwagon effect.mp.

49 framing effect.mp.

50 overconfidence.mp.

51 order effect.mp.

52 recency bias*.mp.

53 26 or 27 or 28 or 29 or 30 or 31 or 32 or 33 or 34 or 35 or 36 or 37 or 38 or 39 or 40 or 41 or 42 or 43 or 44 or 45 or 46 or 47 or 48 or 49 or 50 or 51 or 52 1058995

54 decision making.mp.

55 decision.mp.

56 diagnostic.mp.

57 metacognition.mp.

58 54 or 55 or 56 or 57

59 53 or 58

60 53 and 58

61 teach*.mp.

62 educat*.mp.

63 University.mp.

64 assesment.mp.

65 examination.mp.

66 test*.mp.

67 tertiary educat*.mp.

68 61 or 62 or 63 or 64 or 65 or 66 or 67

69 25 and 59 and 68

70 limit 69 to (2227 educational measurement or 2340 cognitive processes or 3100 personality psychology or 3120 personality traits & processes or 3410 professional education & training or 3530 curriculum & programs & teaching methods)

71 limit 70 to randomized controlled trial

72 limit 71 to (dissertation or journal article)

73 limit 72 to (meta analysis or "systematic review")

74 limit 73 to english language

75 limit 74 to human

76 limit 75 to ("review articles" or systematic reviews or adaptive clinical trial or case reports or clinical study or comparative study or controlled clinical trial or evaluation study or journal article or meta analysis or observational study or pragmatic clinical trial or randomized controlled trial or "systematic review")
