# Supplementary material for: Educational strategies in the health professions to mitigate cognitive and implicit bias impact on decision making: a scoping review
Source: BMC Med Educ. 2023 Jun 20;23:455. doi: 10.1186/s12909-023-04371-5 (PMC10280953; doi:10.1186/s12909-023-04371-5)
Supplement: Supplementary file 2 — Additional file 2: Supplementary Table 1. Exclude Articles. [file 12909_2023_4371_MOESM2_ESM.docx]

Supplementary Table 1: Exclude Articles

| **Full reference** | **Article Title** | **Reason for exclusion** |
| --- | --- | --- |
| Altabbaa et al (2019) [1] | A simulation-based approach to training in heuristic clinical decision-making | Exclusion reason: Incorrect target population of interest |
| Callender et al. (2016) [2] | Improving metacognition in the classroom through instruction, training, and feedback | Exclusion reason: Incorrect target population of interest |
| Godsil et al. (2014) [3] | Addressing implicit bias, racial anxiety, and stereotype threat in education and health care | Exclusion reason: Incorrect target population of interest |
| Gonzalez et al. (2018) [4] | How to make or break implicit bias instruction: implications for curriculum development | Exclusion reason: Incorrect target population of interest |
| Hall et al. (2015) [5] | Implicit racial/ethnic bias among health care professionals and its influence on health care outcomes: a systematic review | Exclusion reason: Incorrect target population of interest |
| Harrison-Bernard et al. (2020) [6] | Knowledge gains in a professional development workshop on diversity, equity, inclusion, and implicit bias in academia | Exclusion reason: Incorrect target population of interest |
| Hunzeker & Amin (2016) [7] | Teaching Cognitive Bias in a Hurry: Single-Session Workshop Approach for Psychiatry Residents and Students | Exclusion reason: Incorrect target population of interest |
| McDowell et al. (2020) [8] | Strategies to mitigate clinician implicit bias against sexual and gender minority patients | Exclusion reason: Incorrect target population of interest |
| O'Sullivan & Schofield (2019) | A cognitive forcing tool to mitigate cognitive bias - a randomised control trial | Exclusion reason: Incorrect target population of interest |
| Perdomo et al. (2019) [9] | Health equity rounds: an interdisciplinary case conference to address implicit bias and structural racism for faculty and trainees | Exclusion reason: Incorrect target population of interest |
| Prakash et al. (2017) [10] | Immersive high fidelity simulation of critically ill patients to study cognitive errors: a pilot study | Exclusion reason: Incorrect target population of interest |
| Reilly et al. (2013) [11] | Teaching about how doctors think: a longitudinal curriculum in cognitive bias and diagnostic error for residents | Exclusion reason: Incorrect target population of interest |
| Rynders (2019) [12] | Battling implicit bias in the IDEA to advocate for African American students with disabilities | Exclusion reason: Incorrect target population of interest |
| Stroumsa et al. (2019) [13] | Transphobia rather than education predicts provider knowledge of transgender health care | Exclusion reason: Incorrect target population of interest |
| Sukhera et al. (2018) [14] | The actual versus idealized self: Exploring responses to feedback about implicit bias in health professionals | Exclusion reason: Incorrect target population of interest |
| Sukhera et al. (2018) [15] | Adaptive reinventing: implicit bias and the co-construction of social change | Exclusion reason: Incorrect target population of interest |
| Sukhera et al. (2018) [16] | Striving while accepting: Exploring the relationship between identity and implicit bias recognition and management | Exclusion reason: Incorrect target population of interest |
| Sukhera et al. (2019) [17] | Implicit bias and the feedback paradox: exploring how health professionals engage with feedback while questioning its credibility | Exclusion reason: Incorrect target population of interest |
| Sukhera et al. (2019) [18] | The Implicit Association Test in health professions education: A meta-narrative review | Exclusion reason: Incorrect target population of interest |
| Westerberg (2016) [19] | Understanding and dealing with implicit bias and discipline in early care and education | Exclusion reason: Incorrect target population of interest |
| Almashat et al (2008) [20] | Framing effect debiasing in medical decision making | Exclusion reason: Incorrect concept. |
| Avant, et al. (2018) [21] | Qualitative analysis of student pharmacists’ reflections of Harvard’s race implicit association test. | Exclusion reason: Incorrect concept. |
| Braun et al (2019) [22] | Scaffolding clinical reasoning of medical students with virtual patients: effects on diagnostic accuracy, efficiency, and errors | Exclusion reason: Incorrect concept. |
| Burt & Corbridge (2018) [23] | Teaching Diagnostic Reasoning: A Review of Evidence-Based Interventions | Send to 3rd person for consideration.  Exclusion reason: Incorrect concept. |
| Capers IV, (2020) [24] | How clinicians and educators can mitigate implicit bias in patient care and candidate selection in medical education. | Exclusion reason: Incorrect concept. |
| Carabez et al. (2015) [25] | Nursing students' perceptions of their knowledge of lesbian, gay, bisexual, and transgender issues: effectiveness of a multi-purpose assignment in a public health nursing class | Exclusion reason: Incorrect concept |
| Desy, et al. (2019) [26] | How can we reduce bias during an academic assessment reappraisal? | Exclusion reason: Incorrect concept. |
| Dixon-Woods et al. (2002) | Teaching and learning about human sexuality in undergraduate medical education | Exclusion reason: Incorrect concept. |
| Gonzalez et al. (2018) [27] | Patient perspectives on racial and ethnic implicit bias in clinical encounters: Implications for curriculum development. | Exclusion reason: Incorrect concept. |
| Gonzalez et al. (2019) [28] | A Qualitative Study of New York Medical Student Views on Implicit Bias Instruction: Implications for Curriculum Development. | Exclusion reason: Incorrect concept. |
| Harris et al. (2016) [29] | Development and testing of study tools and methods to examine ethnic bias and clinical decision-making among medical students in New Zealand: The Bias and Decision-Making in Medicine (BDMM) study | Exclusion reason: Incorrect concept. |
| Hernandez (2018) [30] | Medical students' implicit bias and the communication of norms in medical education. | Exclusion reason: Incorrect concept. |
| Issac & Behar-Horenstein (2016) [31] | Impact of interviews on heterosexual students' expression of cultural competency. | Exclusion reason: Incorrect concept. |
| Johnson et al. (2015) [32] | Student-Initiated Sexual Health Selective as a Curricular Tool | Exclusion reason: Incorrect concept. |
| Kassirer (2010) [33] | Teaching clinical reasoning: case-based and coached. | Exclusion reason: Incorrect concept. |
| Keiller & Hanekom (2014) [34] | Strategies to increase clinical reasoning and critical thinking in physiotherapy education. | Exclusion reason: Incorrect concept. |
| Kelley et al. (2008) [35] | A critical intervention in lesbian, gay, bisexual, and transgender health: knowledge and attitude outcomes among second-year medical students | Exclusion reason: Incorrect concept. |
| Klayman & Brown (1993) [36] | Debias the environment instead of the judge: An alternative approach to reducing error in diagnostic (and other) judgment | Exclusion reason: Incorrect concept. |
| Klein et al. (2019) [37] | Fostering medical students' clinical reasoning by learning from errors in clinical case vignettes: effects and conditions of additional prompting procedures to foster self-explanations | Exclusion reason: Incorrect concept. |
| Lambe et al. (2018) [38] | Guided Reflection Interventions Show No Effect on Diagnostic Accuracy in Medical Students | Send to 3rd person for consideration.  Exclusion reason: Incorrect concept. |
| Maina et al. (2018) [39] | A decade of studying implicit racial/ethnic bias in healthcare providers using the implicit association test | Exclusion reason: Incorrect concept. |
| McCormick & Seta (2012) [40] | Lateralized goal framing: How selective presentation impacts message effectiveness | Exclusion reason: Incorrect concept. |
| Myung et al. (2013) [41] | Effect of enhanced analytic reasoning on diagnostic accuracy: a randomized controlled study | Exclusion reason: Incorrect concept. |
| Phelan et al. (2015) [42] | The mixed impact of medical school on medical students' implicit and explicit weight bias | Exclusion reason: Incorrect concept. |
| Reinsch et al. (2020) [43] | Student evaluations and the problem of implicit bias | Exclusion reason: Incorrect concept. |
| Richie & Josephson (2018) [44] | Quantifying heuristic bias: Anchoring, availability, and representativeness | Exclusion reason: Incorrect concept. |
| Sanders & McHugh (2021) [45] | Pre-clerkship medical students' experiences and perspectives of System 1 and System 2 thinking: A qualitative study | Exclusion reason: Incorrect concept. |
| Strong & Folse (2015) [46] | Assessing undergraduate nursing students' knowledge, attitudes, and cultural competence in caring for lesbian, gay, bisexual, and transgender patients | Exclusion reason: Incorrect concept. |
| Taris et al. (2001) [47] | Job stress, job strain, and psychological withdrawal among Dutch university staff: towards a dual-process model for the effects of occupational stress | Exclusion reason: Incorrect concept. |
| Thomas & Valli (2006) [48] | Levels of occupational stress in doctors working in a South African public-sector hospital | Exclusion reason: Incorrect concept. |
| Wallsten (1981) [49] | Physician and medical student bias in evaluating diagnostic information | Exclusion reason: Incorrect concept. |
| Neitzel (2018) [50] | Research to practice: Understanding the role of implicit bias in early childhood disciplinary practices | Exclusion reason: Incorrect context |
| Bhatti (2018) [51] | Cognitive bias in clinical practice - nurturing healthy skepticism among medical students | Exclusion reason: Wrong publication type (e.g. opinion piece, editorial); |
| Boscardin (2015) [52] | Reducing implicit bias through curricular interventions | Exclusion reason: Wrong publication type (e.g. opinion piece, editorial); |
| Bou Khalil et al. (2020) [53] | Teaching the use of framing and decontextualization to address context-based bias in psychiatry | Exclusion reason: Wrong publication type (e.g. opinion piece, editorial); |
| Boysen (2010) [54] | Integrating implicit bias into counselor education | Exclusion reason: Wrong publication type (e.g. opinion piece, editorial); |
| Buchs & Mulitalo (2016) [55] | Implicit bias: An opportunity for physician assistants to mindfully reduce health care disparities | Exclusion reason: Wrong publication type (e.g. opinion piece, editorial); |
| Burgess et al. (2017) [56] | Mindfulness practice: A promising approach to reducing the effects of clinician implicit bias on patients | Exclusion reason: Wrong publication type (e.g. opinion piece, editorial); |
| Byrne & Tanesini (2015) [57] | Instilling new habits: addressing implicit bias in healthcare professionals | Exclusion reason: Wrong publication type (e.g. opinion piece, editorial); |
| Chapman et al. (2013) [58] | Physicians and implicit bias: how doctors may unwittingly perpetuate health care disparities | Exclusion reason: Wrong publication type (e.g. opinion piece, editorial); |
| Cooke (2017) [59] | Implicit bias in academic medicine: #WhatADoctorLooksLike | Exclusion reason: Wrong publication type (e.g. opinion piece, editorial) |
| Crandlemire (2020) [60] | Unconscious bias and the impacts on caring: The role of the clinical nursing instructor | Exclusion reason: Wrong publication type (e.g. opinion piece, editorial); |
| Fallin-Bennett (2015) [61] | Implicit bias against sexual minorities in medicine: cycles of professional influence and the role of the hidden curriculum | Exclusion reason: Wrong publication type (e.g. opinion piece, editorial); |
| Griffith et al. (2020) [62] | Education initiatives in cognitive debiasing to improve diagnostic accuracy in student providers: A scoping review | Exclusion reason Wrong publication type (e.g. opinion piece, editorial); |
| Hagiwara et al. (2020) [63] | A call for grounding implicit bias training in clinical and translational frameworks | Exclusion reason: Wrong publication type (e.g. opinion piece, editorial) |
| Mayfield et al. (2017) [64] | Beyond men, women, or both: a comprehensive, LGBTQ-inclusive, implicit-bias-aware, standardized-patient-based sexual history taking curriculum | Exclusion reason: Wrong publication type (e.g. opinion piece, editorial); |
| Mishra et al. (2017) [65] | Teaching for Reducing Diagnostic Errors | Exclusion reason: Wrong publication type (e.g. opinion piece, editorial); |
| Morris et al. (2019) [66] | Training to reduce LGBTQ-related bias among medical, nursing, and dental students and providers: a systematic review | Exclusion reason: Wrong publication type (e.g. opinion piece, editorial); |
| Plews-Ogan et al. (2020) [67] | Acting Wisely: Eliminating Negative Bias in Medical Education-Part 2: How Can We Do Better? | Exclusion reason: Wrong publication type (e.g. opinion piece, editorial) |
| Sellier et al. (2019) [68] | Debiasing training improves decision making in the field | Exclusion reason: Wrong publication type (e.g. opinion piece, editorial); |
| Sellier et al. (2020) [69] | "Debiasing training improves decision making in the field": Corrigendum | Exclusion reason: Wrong publication type (e.g. opinion piece, editorial); |
| Staats (2016) [70] | Understanding implicit bias: What educators should know | Exclusion reason: Wrong publication type (e.g. opinion piece, editorial); |
| Sukhera & Watling (2018) [71] | A framework for integrating implicit bias recognition into health professions education | Exclusion reason: Wrong publication type (e.g. opinion piece, editorial); |
| Sukhera et al. (2020) [72] | Implicit bias in health professions: from recognition to transformation | Exclusion reason: Wrong publication type (e.g. opinion piece, editorial); |
| Thomas & Booth-McCoy, (2020) [73] | Blackface, implicit bias, and the informal curriculum: shaping the healthcare workforce, and improving health | Exclusion reason: Wrong publication type (e.g. opinion piece, editorial) |
| Tsai & Crawford-Roberts (2017) [74] | A call for critical race theory in medical education | Exclusion reason: Wrong publication type (e.g. opinion piece, editorial); |
| Zestcott et al. (2016) [75] | Examining the presence, consequences, and reduction of implicit bias in health care: a narrative review | Exclusion reason: Wrong publication type (e.g. opinion piece, editorial); |

**Reference List**

1. Altabbaa G, Raven AD, Laberge J. A simulation-based approach to training in heuristic clinical decision-making. Diagnosis. 2019;6(2):91-9.

2. Callender AA, Franco-Watkins AM, Roberts AS. Improving metacognition in the classroom through instruction, training, and feedback. Metacognition and Learning. 2016;11(2):215-35.

3. Godsil RD, Tropp LR, Goff PA, Powell JA. Addressing implicit bias, racial anxiety, and stereotype threat in education and health care. The Science of Equality. 2014;1:1-90.

4. Gonzalez CM, Garba RJ, Liguori A, Marantz PR, McKee MD, Lypson ML. How to make or break implicit bias instruction: Implications for curriculum development. Academic medicine: journal of the Association of American Medical Colleges. 2018;93(11):S74.

5. Hall WJ, Chapman MV, Lee KM, Merino YM, Thomas TW, Payne BK, et al. Implicit racial/ethnic bias among health care professionals and its influence on health care outcomes: A systematic review. American journal of public health. 2015;105(12):e60-e76.

6. Harrison-Bernard LM, Augustus-Wallace AC, Souza-Smith FM, Tsien F, Casey GP, Gunaldo TP. Knowledge gains in a professional development workshop on diversity, equity, inclusion, and implicit bias in academia. Adv Physiol Educ. 2020;44(3):286-94.

7. Hunzeker A, Amin R. Teaching cognitive bias in a hurry: Single-session workshop approach for psychiatry residents and students. MedEdPORTAL publ. 2016;12:10451.

8. McDowell MJ, Goldhammer H, Potter JE, Keuroghlian AS. Strategies to mitigate clinician implicit bias against sexual and gender minority patients. Psychosomatics. 2020;61(6):655-61.

9. Perdomo J, Tolliver D, Hsu H, He Y, Nash KA, Donatelli S, et al. Health equity rounds: An interdisciplinary case conference to address implicit bias and structural racism for faculty and trainees. MedEdPORTAL. 2019;15:10858.

10. Prakash S, Bihari S, Need P, Sprick C, Schuwirth L. Immersive high fidelity simulation of critically ill patients to study cognitive errors: A pilot study. BMC Med Educ. 2017;17(1):36.

11. Reilly JB, Ogdie AR, Von Feldt JM, Myers JS. Teaching about how doctors think: A longitudinal curriculum in cognitive bias and diagnostic error for residents. BMJ Quality & Safety. 2013;22(12):1044-50.

12. Rynders D. Battling implicit bias in the idea to advocate for african american students with disabilities. Touro L Rev. 2019;35:461.

13. Stroumsa D, Shires DA, Richardson CR, Jaffee KD, Woodford MR. Transphobia rather than education predicts provider knowledge of transgender health care. Medical education. 2019;53(4):398-407.

14. Sukhera J, Milne A, Teunissen PW, Lingard L, Watling C. The actual versus idealized self: Exploring responses to feedback about implicit bias in health professionals. Acad Med. 2018;93(4):623-9.

15. Sukhera J, Milne A, Teunissen PW, Lingard L, Watling C. Adaptive reinventing: Implicit bias and the co-construction of social change. Adv Health Sci Educ Theory Pract. 2018;23(3):587-99.

16. Sukhera J, Wodzinski M, Teunissen PW, Lingard L, Watling C. Striving while accepting: Exploring the relationship between identity and implicit bias recognition and management. Acad Med. 2018;93(11S):S82-S8.

17. Sukhera J, Wodzinski M, Milne A, Teunissen PW, Lingard L, Watling C. Implicit bias and the feedback paradox: Exploring how health professionals engage with feedback while questioning its credibility. Acad Med. 2019;94(8):1204-10.

18. Sukhera J, Wodzinski M, Rehman M, Gonzalez CM. The implicit association test in health professions education: A meta-narrative review. Perspect Med Educ. 2019;8(5):267-75.

19. Westerberg D. Understanding and dealing with implicit bias and discipline in early care and education. The Brown University Child and Adolescent Behavior Letter. 2016;32(10):1-6.

20. Almashat S, Ayotte B, Edelstein B, Margrett J. Framing effect debiasing in medical decision making. Patient Educ Couns. 2008;71(1):102-7.

21. Avant ND, Weed E, Connelly C, Hincapie AL, Penm J. Qualitative analysis of student pharmacists’ reflections of harvard’s race implicit association test. Currents in Pharmacy Teaching and Learning. 2018;10(5):611-7.

22. Braun LT, Borrmann KF, Lottspeich C, Heinrich DA, Kiesewetter J, Fischer MR, et al. Scaffolding clinical reasoning of medical students with virtual patients: Effects on diagnostic accuracy, efficiency, and errors. Diagnosis (Berl). 2019;6(2):137-49.

23. Burt L, Corbridge S. Teaching diagnostic reasoning: A review of evidence-based interventions. Int. 2018;15(1):02.

24. Capers IV Q. How clinicians and educators can mitigate implicit bias in patient care and candidate selection in medical education. ATS sch. 2020;1(3):211-7.

25. Carabez R, Pellegrini M, Mankovitz A, Eliason MJ, Dariotis WM. Nursing students' perceptions of their knowledge of lesbian, gay, bisexual, and transgender issues: Effectiveness of a multi-purpose assignment in a public health nursing class. J Nurs Educ. 2015;54(1):50-3.

26. Desy J, Coderre S, Davis M, Cusano R, McLaughlin K. How can we reduce bias during an academic assessment reappraisal? Medical Teacher. 2019;41(11):1315-8.

27. Gonzalez CM, Deno ML, Kintzer E, Marantz PR, Lypson ML, McKee MD. Patient perspectives on racial and ethnic implicit bias in clinical encounters: Implications for curriculum development. Patient education and counseling. 2018;101(9):1669-75.

28. Gonzalez CM, Deno ML, Kintzer E, Marantz PR, Lypson ML, McKee MD. A qualitative study of new york medical student views on implicit bias instruction: Implications for curriculum development. J Gen Intern Med. 2019;34(5):692-8.

29. Harris R, Cormack D, Curtis E, Jones R, Stanley J, Lacey C. Development and testing of study tools and methods to examine ethnic bias and clinical decision-making among medical students in new zealand: The bias and decision-making in medicine (bdmm) study. BMC Med Educ. 2016;16:173.

30. Hernandez R. Medical students' implicit bias and the communication of norms in medical education. Teaching and Learning in Medicine. 2018;30(1):112-7.

31. Issac C, Behar-Horenstein L. Impact of interviews on heterosexual students' expression of cultural competency. The Qualitative Report. 2016;21(10):13.

32. Johnson K, Rullo J, Faubion S. Student-initiated sexual health selective as a curricular tool. Sex Med. 2015;3(2):118-27.

33. Kassirer JP. Teaching clinical reasoning: Case-based and coached. Acad Med. 2010;85(7):1118-24.

34. Keiller L, Hanekom SD. Strategies to increase clinical reasoning and critical thinking in physiotherapy education. South African Journal of Physiotherapy. 2014;70(1):8-12.

35. Kelley L, Chou CL, Dibble SL, Robertson PA. A critical intervention in lesbian, gay, bisexual, and transgender health: Knowledge and attitude outcomes among second-year medical students. Teach Learn Med. 2008;20(3):248-53.

36. Klayman J, Brown K. Debias the environment instead of the judge: An alternative approach to reducing error in diagnostic (and other) judgment. Cognition. 1993;49(1-2):97-122.

37. Klein M, Otto B, Fischer MR, Stark R. Fostering medical students' clinical reasoning by learning from errors in clinical case vignettes: Effects and conditions of additional prompting procedures to foster self-explanations. Adv Health Sci Educ Theory Pract. 2019;24(2):331-51.

38. Lambe KA, Hevey D, Kelly BD. Guided reflection interventions show no effect on diagnostic accuracy in medical students. Front Psychol. 2018;9:2297.

39. Maina IW, Belton TD, Ginzberg S, Singh A, Johnson TJ. A decade of studying implicit racial/ethnic bias in healthcare providers using the implicit association test. Social Science & Medicine. 2018;199:219-29.

40. McCormick M, Seta JJ. Lateralized goal framing: How selective presentation impacts message effectiveness. Journal of Health Psychology. 2012;17(8):1099-109.

41. Myung SJ, Kang SH, Phyo SR, Shin JS, Park WB. Effect of enhanced analytic reasoning on diagnostic accuracy: A randomized controlled study. Medical Teacher. 2013;35(3):248-50.

42. Phelan SM, Puhl RM, Burke SE, Hardeman R, Dovidio JF, Nelson DB, et al. The mixed impact of medical school on medical students' implicit and explicit weight bias. Medical Education. 2015;49(10):983-92.

43. Reinsch RW, Goltz SM, Hietapelto AB. Student evaluations and the problem of implicit bias. JC & UL. 2020;45:114.

44. Richie M, Josephson S. Quantifying heuristic bias: Anchoring, availability, and representativeness. Teaching and Learning in Medicine. 2018;30(1):67-75.

45. Sanders W, McHugh D. Pre-clerkship medical students' experiences and perspectives of system 1 and system 2 thinking: A qualitative study. Education Sciences Vol 11(2), 2021, ArtID 34. 2021;11(2).

46. Strong KL, Folse VN. Assessing undergraduate nursing students' knowledge, attitudes, and cultural competence in caring for lesbian, gay, bisexual, and transgender patients. J Nurs Educ. 2015;54(1):45-9.

47. Taris TW, Schreurs PJG, van Iersel-van Silfhout IJ. Job stress, job strain, and psychological withdrawal among dutch university staff: Towards a dual-process model for the effects of occupational stress. Work & Stress. 2001;15(4):283-96.

48. Thomas LS, Valli A. Levels of occupational stress in doctors working in a south african public-sector hospital. South African Medical Journal. 2006;96(11):1162-8.

49. Wallsten TS. Physician and medical student bias in evaluating diagnostic information. Med Decis Making. 1981;1(2):145-64.

50. Neitzel J. Research to practice: Understanding the role of implicit bias in early childhood disciplinary practices. Journal of Early Childhood Teacher Education. 2018;39(3):232-42.

51. Bhatti A. Cognitive bias in clinical practice–nurturing healthy skepticism among medical students. Advances in Medical Education and Practice. 2018;9:235.

52. Boscardin CK. Reducing implicit bias through curricular interventions. Springer; 2015. p. 1726-8.

53. Bou Khalil R, Sleilaty G, El-Khoury J, Nemr E. Teaching the use of framing and decontextualization to address context-based bias in psychiatry. Asian J Psychiatr. 2020;54:102276.

54. Boysen GA. Integrating implicit bias into counselor education. Counselor Education and Supervision. 2010;49(4):210-27.

55. Buchs S, Mulitalo K. Implicit bias: An opportunity for physician assistants to mindfully reduce health care disparities. The Journal of Physician Assistant Education. 2016;27(4):193-5.

56. Burgess DJ, Beach MC, Saha S. Mindfulness practice: A promising approach to reducing the effects of clinician implicit bias on patients. Patient Education and Counseling. 2017;100(2):372-6.

57. Byrne A, Tanesini A. Instilling new habits: Addressing implicit bias in healthcare professionals. Adv Health Sci Educ Theory Pract. 2015;20(5):1255-62.

58. Chapman EN, Kaatz A, Carnes M. Physicians and implicit bias: How doctors may unwittingly perpetuate health care disparities. J Gen Intern Med. 2013;28(11):1504-10.

59. Cooke M. Implicit bias in academic medicine:# whatadoctorlookslike. JAMA Internal Medicine. 2017;177(5):657-8.

60. Crandlemire LA. Unconscious bias and the impacts on caring: The role of the clinical nursing instructor. International Journal for Human Caring. 2020;24(2):84-91.

61. Fallin-Bennett K. Implicit bias against sexual minorities in medicine: Cycles of professional influence and the role of the hidden curriculum. Acad Med. 2015;90(5):549-52.

62. Griffith PB, Doherty C, Smeltzer SC, Mariani B. Education initiatives in cognitive debiasing to improve diagnostic accuracy in student providers: A scoping review. Journal of the American Association of Nurse Practitioners. 2020;33(11):862-71.

63. Hagiwara N, Kron FW, Scerbo MW, Watson GS. A call for grounding implicit bias training in clinical and translational frameworks. The Lancet. 2020;395(10234):1457-60.

64. Mayfield JJ, Ball EM, Tillery KA, Crandall C, Dexter J, Winer JM, et al. Beyond men, women, or both: A comprehensive, lgbtq-inclusive, implicit-bias-aware, standardized-patient-based sexual history taking curriculum. MedEdPORTAL. 2017;13:10634.

65. Mishra D, Gupta P, Singh T. Teaching for reducing diagnostic errors. Indian Pediatr. 2017;54(1):37-45.

66. Morris M, Cooper RL, Ramesh A, Tabatabai M, Arcury TA, Shinn M, et al. Training to reduce lgbtq-related bias among medical, nursing, and dental students and providers: A systematic review. BMC Med Educ. 2019;19(1):1-13.

67. Plews-Ogan ML, Bell TD, Townsend G, Canterbury RJ, Wilkes DS. Acting wisely: Eliminating negative bias in medical education-part 2: How can we do better? Acad Med. 2020;95(12S Addressing Harmful Bias and Eliminating Discrimination in Health Professions Learning Environments):S16-S22.

68. Sellier A-L, Scopelliti I, Morewedge CK. Debiasing training improves decision making in the field. Psychol Sci. 2019;30(9):1371-9.

69. Sellier A-L, Scopelliti I, Morewedge CK. "Debiasing training improves decision making in the field": Corrigendum. Psychol Sci. 2020;31(6):762.

70. Staats C. Understanding implicit bias: What educators should know. American Educator. 2016;39(4):29.

71. Sukhera J, Watling C. A framework for integrating implicit bias recognition into health professions education. Acad Med. 2018;93(1):35-40.

72. Sukhera J, Watling CJ, Gonzalez CM. Implicit bias in health professions: From recognition to transformation. Acad Med. 2020;95(5):717-23.

73. Thomas B, Booth-McCoy AN. Blackface, implicit bias, and the informal curriculum: Shaping the healthcare workforce, and improving health. Journal of the National Medical Association. 2020;112(5):533-40.

74. Tsai J, Crawford-Roberts A. A call for critical race theory in medical education. Acad Med. 2017;92(8):1072-3.

75. Zestcott CA, Blair IV, Stone J. Examining the presence, consequences, and reduction of implicit bias in health care: A narrative review. Group Processes & Intergroup Relations. 2016;19(4):528-42.
